# Microbial Community Response on Wastewater Discharge in Boreal Lake Sediments

**DOI:** 10.3389/fmicb.2017.00750

**Published:** 2017-04-25

**Authors:** Jatta Saarenheimo, Sanni L. Aalto, Antti J. Rissanen, Marja Tiirola

**Affiliations:** ^1^Department of Biological and Environmental Science, University of JyväskyläJyväskylä, Finland; ^2^Laboratory of Chemistry and Bioengineering, Tampere University of TechnologyTampere, Finland

**Keywords:** AOA, AOB, community composition, denitrification, nitrification, *nirS*, *nirK*, *nosZ*

## Abstract

Despite high performance, municipal wastewater treatment plants (WWTPs) still discharge significant amounts of organic material and nitrogen and even microbes into the receiving water bodies, altering physico-chemical conditions and microbial functions. In this study, we examined how nitrified wastewater affects the microbiology of boreal lake sediments. Microbial community compositions were assessed with next generation sequencing of the 16S rRNA gene, and a more detailed view on nitrogen transformation processes was gained with qPCR targeting on functional genes (*nirS, nirK, nosZ*_I_, *nosZ*_II_, *amoA*_archaea_, and *amoA*_bacteria_). In both of the two studied lake sites, the microbial community composition differed significantly between control point and wastewater discharge point, and a gradual shift toward natural community composition was seen downstream following the wastewater gradient. SourceTracker analysis predicted that ∼2% of sediment microbes were of WWTP-origin on the study site where wastewater was freely mixed with the lake water, while when wastewater was specially discharged to the sediment surface, ∼6% of microbes originated from WWTP, but the wastewater-influenced area was more limited. In nitrogen transformation processes, the ratio between nitrifying archaea (AOA) and bacteria (AOB) was affected by wastewater effluent, as the AOA abundance decreased from the control point (AOA:AOB 28:1 in Keuruu, 11:1 in Petäjävesi) to the wastewater-influenced sampling points, where AOB dominated (AOA:AOB 1:2–1:15 in Keuruu, 1:3–1:19 in Petäjävesi). The study showed that wastewater can affect sediment microbial community through importing nutrients and organic material and altering habitat characteristics, but also through bringing wastewater-originated microbes to the sediment, and may thus have significant impact on the freshwater biogeochemistry, especially in the nutrient-poor boreal ecosystems.

## Introduction

Municipal wastewater treatment plants (WWTPs) are important point sources of nutrients, especially nitrogen (N), and organic material, altering biogeochemical processes in the receiving ecosystems (e.g., lake or river) (Lofton et al., 2007; Carey and Migliaccio, 2009). Wastewater can modify the function and composition of the sediment microbial community by altering physico-chemical habitat characteristics (Wakelin et al., 2008; Lu and Lu, 2014; Rahm et al., 2016). Wastewater may reduce microbial population size and diversity, favoring certain functional groups and leading to biotic homogenization (Drury et al., 2013; Lu and Lu, 2014). In addition to indirectly modifying microbial community through habitat characteristics, wastewater can bring in new microbes, which may change prevailing community composition and function (Dang et al., 2010; Chahinian et al., 2012). However, the capability of WWTP-originating microbes to establish and compete in natural aquatic systems has been questioned, as previous studies have mainly focused on lotic dynamic systems, e.g., rivers (e.g., Garnier et al., 1992; Wakelin et al., 2008; Drury et al., 2013; Rahm et al., 2016). Although previous studies have successfully demonstrated that wastewater can modify the microbial communities in the receiving waterbodies, the knowledge on underlying mechanisms and their relative importance is still poor. In addition, previous studies were mainly done in temperate areas, whereas the effect of wastewater on microbial communities of nutrient poor aquatic environments in the boreal zone is currently unknown.

The excess wastewater nitrogen can be removed by microbiological processes in the receiving waterbody. These processes take mainly place within the top layers of sediment, where the microbial activity is highest. Depending on the form in which N is released from WWTP, it can be either converted from ammonium (NH_4_^+^) to nitrate (NO_3_^-^) in nitrification, or reduced from nitrate through intermediate steps to nitrogen gas (N_2_) in denitrification. These processes can also be linked, and form an important pathway removing biologically reactive nitrogen load from the ecosystems (Seitzinger et al., 2006). The key step of nitrification, ammonium oxidation to nitrite (NO_2_^-^), is driven by ammonia-oxidizing archaea (AOA) and ammonia-oxidizing bacteria (AOB) under oxic conditions. AOA are known to thrive under more stable and N poor conditions, whereas AOB have shown to tolerate fluctuating environmental conditions and outcompete AOA in N rich environments (Laanbroek et al., 2013; Bollmann et al., 2014). Denitrification is an anaerobic process consisting of four reductive steps that are each catalyzed by different enzymes (Zumft, 1997). The most important steps are encoded by analogous genes: the step encoding the reduction of NO_2_ to nitric oxide (NO) by *nirS* and *nirK*, and the step encoding reduction of nitrous oxide (N_2_O) to N_2_ by two clades (I and II) of *nosZ* gene (Sanford et al., 2012; Jones et al., 2014). Previous studies focused on marine or coastal environments have demonstrated that there is a clear niche separation between the two gene analogs (e.g., *nirS* tends to dominate over *nirK*) following environmental gradients (Jones and Hallin, 2010; Wittorf et al., 2016). However, in the boreal lakes, the relative proportions of *nirS* and *nirK*, as well as *nosZ*_I_ and *nosZ*_II_, have been found to be roughly equal, which has been explained by the high seasonal variation in the environmental factors increasing the diversity of ecological niches (Saarenheimo et al., 2015a).

In addition to N form and supply, other environmental factors, such as oxygen concentration, temperature, organic matter concentration and hydraulic residence time, control the N transforming microbes and their interactions in aquatic environments (e.g., Herrmann et al., 2009; Rissanen et al., 2013; Saarenheimo et al., 2015b; Wittorf et al., 2016). Wastewater, by altering all these factors, could thus strongly affect the genetic potential of nitrification and denitrification in the recipient lakes. Indeed, the community patterns of AOB were affected by wastewater in River Seine (Cébron et al., 2004), but the more detailed knowledge on the interactions between AOA and AOB, or on the denitrification gene abundances in wastewater-influenced lake environments is currently lacking.

In boreal environments, lakes undergo strong seasonal temperature stratification, affecting the mixing of oxygen, but also the fate of the wastewater within the lake. In winter and summer, temperature stratification of the recipient waterbody may cause the heavier wastewater to remain longer in the microbe-rich sediment layer, while in spring and autumn, wastewater is readily mixed with the whole water column. This suggests that the effect of wastewater on microbial communities and processes might follow seasonal patterns in boreal area. To study this, we selected two study sites from Central Finland that differed in the design of the wastewater discharge pipe. In Petäjävesi (Lake Jämsänvesi), wastewater flow followed the natural seasonal mixing patterns. In Keuruu (Lake Keurusselkä), wastewater was spatially allocated with a specially designed perforated pipe to remain in the direct contact with the sediment surface throughout the year. Using microbial data collected from upstream and downstream from these two different wastewater discharge points, we examined whether (1) microbial community composition or genetic N-transformation potential was related to wastewater concentration gradient, (2) the shift in the microbial community and/or genetic potential was linked to environmental factors or N removal processes, and (3) microbes originating from the WWTP were detected in the downstream gradient.

## Materials and Methods

### Description of Study Sites

**FIGURE 1:**
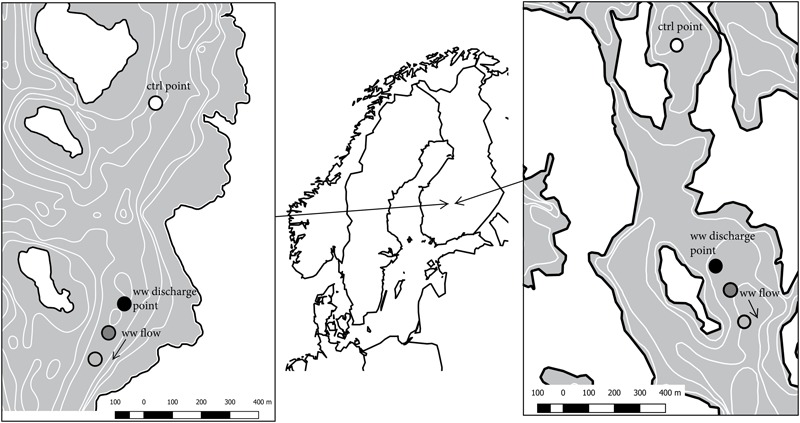
**Study sites and sampling points.** White points represent control sampling points, black points wastewater discharge sampling points, dark gray 100 m downstream sampling points and light gray 200/250 m downstream sampling points. Left: Keuruu, right: Petäjävesi.

Keuruu and Petäjävesi municipal WWTPs are located in Central Finland (**Figure 1**). Both WWTPs are activated sludge plants with nitrification and are required to remove 95% of organic matter and phosphorus and 75–90% of ammonium. In Keuruu (further cited as Keuruu study site), the treated wastewater was discharged through a perforated pipe to the sediment surface of the slowly flowing stream-like upper parts of Lake Keurusselkä (**Figure 1**). The perforated pipe, having 50 holes (diameter 30 mm) on both sides, was attached to the end of the original WWTP discharge pipe at the depth of 9 m in October 2014 as a part of LIFE12 ENV/FI/597 N-SINK -project demonstrations. The main aim was to increase the contact time of wastewater with sediment surface. Lake Keurusselkä is a large humic lake (117 km^2^) and belongs to the large Kokemäenjoki drainage area and its average depth is 6.4 m with a maximum depth of 40 m. The contribution of wastewater effluent (average 2838 m^3^ d^-1^) is minor (0.16%) compared to total lake discharge (1.73 × 10^6^ m^3^ d^-1^).

In Petäjävesi (further cited as Petäjävesi study site), wastewater was discharged to Lake Jämsänvesi, where the wastewater was freely mixed into the water column (depth in the site 4 m). Lake Jämsänvesi is a medium-sized humic lake (0.9 km^2^; **Figure 1**), belonging to Kymijoki drainage area, with average depth of 4.2 m and maximum depth of 27 m. In Petäjävesi, the contribution of wastewater effluent (average 865 m^3^ d^-1^) to the lake total discharge (2.59 × 10^5^ m^3^ d^-1^) is slightly higher (0.33%) than in Keuruu.

### Sample and Data Collection

On Keuruu study site, sediment samples for microbial studies, as well as environmental data, were collected from four different sampling points: control point (800 m upstream), wastewater discharge point, 100 m downstream, and 200 m downstream from the wastewater discharge point (**Figure 1**). Samples were collected during year 2015 at four time points (20 January 2015, 19 May 2015, 11 August 2015, and 20 October 2015). On Petäjävesi study site, sediment samples and environmental data were similarly collected from four sampling points covering control point (900 m upstream), wastewater discharge point, and two sampling points downstream (100 and 250 m) during two sampling occasions (27 January 2015 and 28 May 2015). On both study sites, we collected two samples from each wastewater-influenced sampling point and in addition, we collected three additional samples from the Keuruu control point. Altogether, we had seven control point samples and eight samples from each wastewater-influenced sampling point on Keuruu study site and two control point samples and four samples from each wastewater point on Petäjävesi study site.

Undisturbed sediment cores were collected using Kajak sediment core sampler (KC Denmark A/S). The sediment cores were transported to the laboratory in cold and in dark. For DNA extractions, the uppermost sediment (0–2 cm) was collected within 24 h, at the same time as the process rate measurements were conducted, and stored at -20°C. Oxygen and temperature profiles were measured *in situ* using a portable field meter (YSI model 58, Yellow Springs Instruments). Concurrently collected environmental data included the proportion of organic matter in the sediment (LOI%), as well as nitrate + nitrite (NO_x_^-^) and ammonium (NH_4_^+^) from water above the sediment surface, which were determined as in Rissanen et al. (2011). In Keuruu, sediment denitrification rate (N_2_ production rate) variables were measured using isotope pairing technique (IPT, Nielsen, 1992) following Rissanen et al. (2013), and included Dn% (proportion of coupled nitrification-denitrification), Dw% (proportion of denitrification of the NO_3_^-^ in the water above the sediment), and D14 (total denitrification).

Water samples were collected from both WWTPs in spring 2015 (Keuruu) and autumn 2015 (Petäjävesi) and stored at -20°C. Samples were taken from two treatment phases, from nitrification pool and from the outflow channel.

### Bacterial Community Composition

The sediment samples, as well as water samples from WWTPs, were freeze-dried (Alpha 1-4 LD plus, Martin Christ Gefriertrocknungsanlagen GmbH) and DNA was extracted from 0.2 g of dried sediment using a PowerLyzer PowerSoil DNA extraction kit (MoBio Laboratories, Inc.). Altogether 51 DNA samples were included, covering 32 samples from Keuruu site, 14 samples from Petäjävesi site, 3 samples from the Keuruu WWTP, and 2 samples from the Petäjävesi WWTP.

Changes in the microbial community composition, richness, and diversity of organisms harboring the 16S rRNA gene were studied with next generation sequencing. The DNA templates were PCR amplified by using bacterial 16S rRNA gene primers 27F and 338R following the protocol described in Saarenheimo et al. (2016), with the exception of using Maxima SYBR Green/Fluorescein Master Mix (ThermoFisher) instead of Phusion HotStartII (buffer and polymerase) in all the PCR reactions. Sequencing was conducted with the Ion Torrent Personal Genome Machine (PGM, ThermoFisher) using Ion Sequencing 400 kit and Ion 314 Chip. Analysis of 16S rRNA gene sequences was done using Mothur (Schloss et al., 2009). Sequences shorter than 200 bp, low-quality sequences with more than one mismatch in barcode/primer sequences, or with homopolymers longer than eight nucleotides, as well as barcodes, primers and chimeras were removed. In addition, a 10-bp sliding window with an average quality score 20 was used. A final total of 190142 reads was obtained. Sequences were aligned against Silva reference alignment. Chimeric sequences, denoted using Mothur’s implementation of Uchime (Edgar et al., 2011), were removed from each library. The sequence dataset was re-sampled with command ‘subsample’ in Mothur to gain equal numbers of sequences in each sample. To assign reads to operational taxonomic units (OTUs), a 97% sequence similarity cut-off was used. Singleton OTUs were removed from further analysis. Sequences were classified into taxonomies using Silva.nr_v123 taxonomy files. When analyzing only Keuruu site dataset, 2114 sequences per sample were included, and when analyzing the whole dataset (Keuruu + Petäjävesi + WWTPs), 714 sequences per sample were included. The Ion Torrent sequences have been deposited in the NCBI Short Read Archive under accession number SRP087742.

### Quantitative PCR (qPCR) of N Cycle Specific Genes

Genetic potential of the two essential N transformation processes, denitrification and nitrification, were assessed by targeting functional genes using the Maxima SYBR Green/Fluorescein Master Mix (ThermoFisher) for qPCR. Archaeal and bacterial nitrification genes were investigated using previously described protocols for *amoA*_arch_ (Francis et al., 2005) and *amoA*_bac_ (Rotthauwe et al., 1997). For denitrification, genes of two reduction steps were studied using *nirS* and *nirK* and *nosZ* cladeI and cladeII. Primers are described in Saarenheimo et al., (2015a). All qPCR amplifications and fluorescent data collections were carried out with a Bio-Rad CFX96 thermal cycler (Bio-Rad Laboratorios) in a final reaction mixture of 0.5 μM of each primer for the selected target gene (except for nosZII with which 1 μM concentration was used), 12.5 μl Maxima SYBR Green qPCR Master Mix (ThermoFisher), 1 μl of DNA (10 ng or 5 ng), and PCR-grade water to yield a total volume of 25 μl. Two replicate qPCR amplifications were performed for each sample and each sample was measured with DNA template amounts of 10 and 5 ng. Partial 16S rRNA gene was used as a reference gene. Amplification conditions for 16S rRNA, *nirS, nirK, nosZ*_I_, and *nosZ*_II_ genes as well as the standard curve construction were as described in Saarenheimo et al. (2015a). The PCR procedure for *amoA*_arch_ included an initial denaturation step at 95°C for 10 min and 40 cycles of amplification (95°C for 30 s, 53°C for 30 s, and 72°C for 40 s). Finally, an increase of 0.5°C s-1 from 65 to 95°C was performed to obtain the melting curve analysis of PCR products. The thermal cycling conditions for *amoA*_bac_ were the same as for *amoA*_arch_, except that the annealing temperature was 56°C.

### Statistical Analysis

The functions from the vegan package (Oksanen et al., 2013) were used to examine bacterial community compositions in Keuruu and Petäjävesi lake sediments and WWTPs. Non-metric multidimensional scaling (NMDS, conducted with metaMDS function) plots calculated based on Bray–Curtis distance matrix were used to visualize dynamics in the community structure (β-diversity) of bacteria (OTUs represented by at least two reads) for total data. Before NMDS, Wisconsin and square-root-transformations were applied to OTU abundance data. For Keuruu and Petäjävesi lake data, the differences in bacterial communities between sampling points were tested separately with permutational multivariate analysis of variance (PERMANOVA; Anderson, 2001; McArdle and Anderson, 2001) using the function “adonis” in vegan. The origin of microbes in wastewater-influenced sampling points was predicted with SourceTracker method (Knights et al., 2011) using R package SourceTracker.

Differences in the functional gene abundances and diversity indexes between Keuruu sampling points were examined with Kruskal–Wallis *H*-test. In Petäjävesi data, the low number of data points prevented further statistical testing.

For Keuruu, environmental data [oxygen concentration, temperature, inorganic N concentrations (NO_x_^-^, NH_4_^+^), LOI% as well as N transformation rates (D14, Dn%, Dw%)] were fitted to the NMDS ordination using the function “envfit.” In addition, the interactions between functional gene abundances, Chao and Inverse Simpson indexes, environmental factors and process rates were studied with Spearman rank correlation. The Venn diagram created with the VennDiagram package was used to illustrate differences in unique and shared OTUs between the sampling points.

All statistical analyses were conducted using R version 3.1.1 (R Core Team, 2016).

## Results and Discussion

### Environmental Conditions

The inorganic N concentrations (NO_x_^-^, NH_4_^+^) remained rather similar in the WWTP discharge in 2015, except in Keuruu, when the nitrification collapsed in winter, and water with high NH_4_^+^ concentrations was discharged (Supplementary Table S1). There was a strong seasonal variation in NO_x_^-^ and NH_4_^+^ concentrations, oxygen concentration, the proportion of organic matter in the sediment (LOI%), and total denitrification rates (D14) on both study sites (Supplementary Table S1). LOI% was higher and oxygen concentration was lower at the wastewater discharge point in Petäjävesi than in Keuruu (Supplementary Table S1).

### Microbial Community Composition along Wastewater Gradient

**FIGURE 2:**
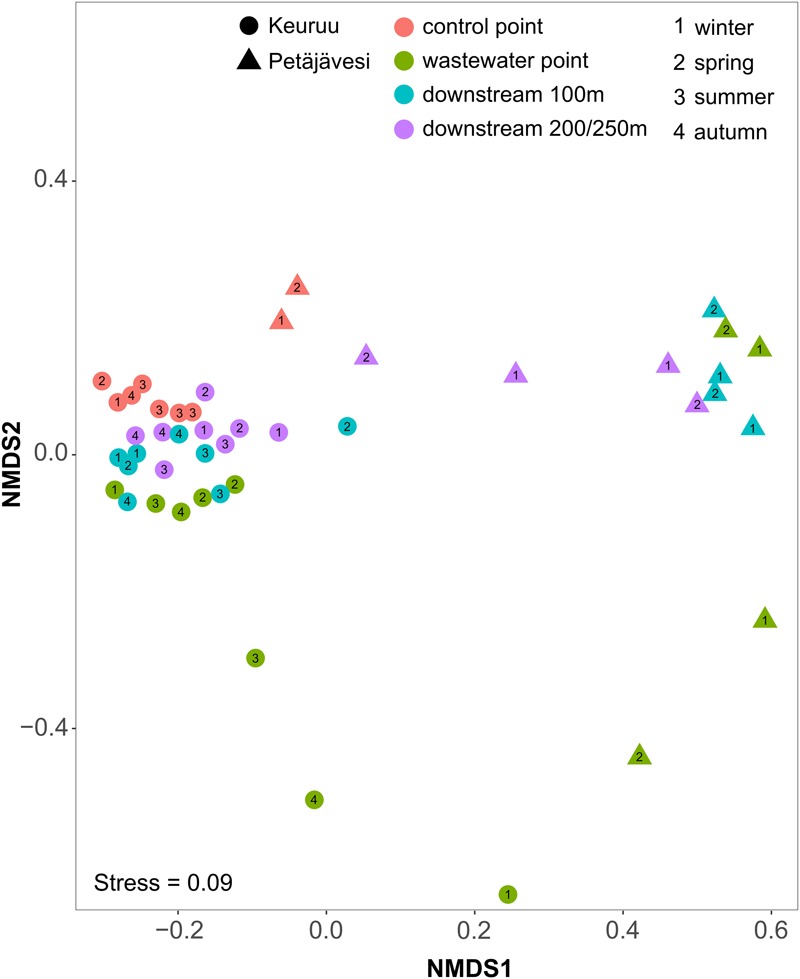
**Non-metric multidimensional scaling (NMDS) of microbial community composition based on Bray–Curtis dissimilarities on Keuruu and Petäjävesi study sites**.

**FIGURE 3:**
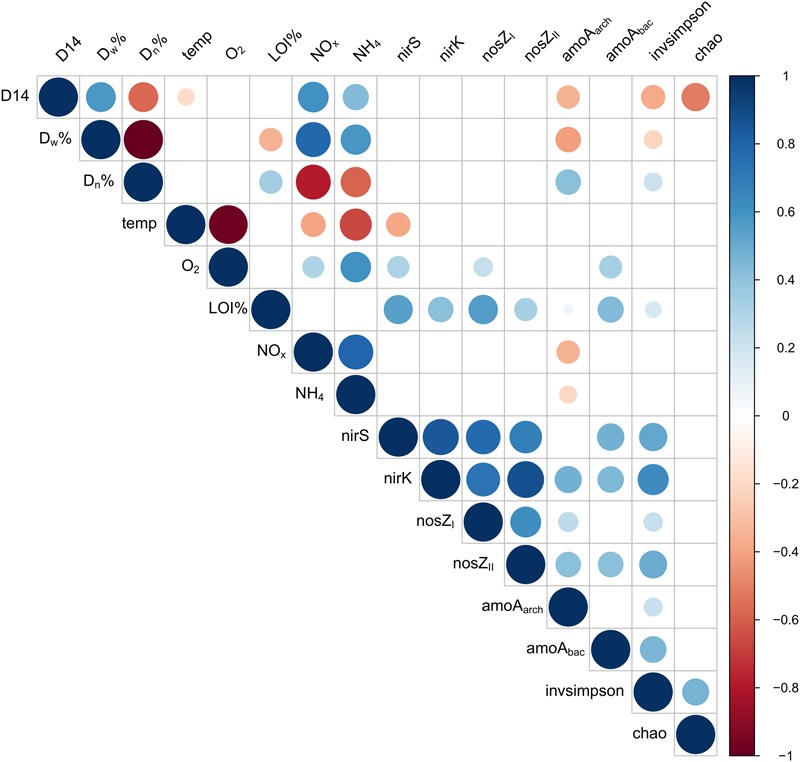
**Correlation plot describing interactions between total denitrification (D14), the proportion of denitrification of the NO_3_^-^ in the water above the sediment (Dw%), the proportion of coupled nitrification-denitrification (Dn%), temperature, oxygen concentration, proportion of organic matter in the sediment (LOI%), nitrate concentration (NO_x_^-^), ammonium concentration (NH_4_^+^), abundances of functional genes (*nirS, nirK, nosZ*_I_, *nosZ*_II_, *amoA*_arch_, *amoA*_bac_) and bacterial diversity (Inverse Simpson index) and richness (chao richness estimator) on Keuruu study site**.

On both of the two study sites, the microbial community composition differed significantly between the upstream control point and the wastewater-influenced sampling points (PERMANOVA, *F* = 1.58, *P* = 0.001 for Keuruu; *F* = 1.43, *P* = 0.003 for Petäjävesi; **Figure 2** and Supplementary Figure S1). The difference in the microbial community structure was greatest between the natural control point and wastewater discharge point, whereas a gradual shift from wastewater-influenced to natural community structure was seen when moving downstream along the wastewater gradient. Although this was seen on both study sites, it was more evident in Keuruu, where wastewater discharge point samples were clearly separated from the downstream and control point samples (Supplementary Figure S1). There, the differences in the microbial community composition were connected to nitrate concentration and measured denitrification process rates, but not to temperature or oxygen (Supplementary Figure S1). In Petäjävesi, the three wastewater-influenced point samples were more similar, being clearly distinct from the control point samples (**Figure 2**). On both study sites, the microbial community varied between the sampling occasions at the wastewater discharge point, whereas at the control and downstream sampling points, the temporal variation in the community composition was low (**Figure 2**). The relationships between bacterial diversity (Inverse Simpson index) and richness (Chao richness estimator), environmental variables and denitrification process rates were studied on Keuruu study site, where both diversity and richness were found to correlate negatively with the total denitrification rate (**Figure 3**). However, neither one was found to be significantly lower at the wastewater-influenced sampling points than at the control point (Supplementary Table S2).

In agreement with the previous studies from river and stream ecosystems (Wakelin et al., 2008; Drury et al., 2013; Lu and Lu, 2014), wastewater altered the sediment microbial community composition on both of our study sites. In Keuruu, the changes in microbial community composition were related to nitrate and denitrification rates, which both followed the wastewater gradient (Supplementary Table S1). In addition, both microbial diversity and richness were lower when total denitrification rate increased. These results indicate that wastewater favored certain functional microbial groups by providing them nutrients and organic material, which led to biotic homogenization, agreeing with the previous studies (e.g., Drury et al., 2013; Lu and Lu, 2014; Atashgahi et al., 2015). Furthermore, the community composition was not driven by seasonal factors (e.g., temperature), but by wastewater composition and discharge volume, as the high variation between the sampling occasions was observed only at the wastewater discharge sampling point on both study sites. If microbial community composition had been controlled by seasonal environmental factors, also other sampling points would have shown a similar temporal variation. In general, these results support our hypothesis that changes in mixing patterns of wastewater with lake water, altering the contact time and area of wastewater with sediment surface, have significant role on regulating the overall effect of wastewater on lake microbial communities. If wastewater is discharged to the sediment surface (as in Keuruu), it has strong yet spatially more restricted impact on microbes, as diffusion distance of nutrients or oxygen or other substances is short as well as turbulence can be high. However, if wastewater is mixed within the whole water column (as in Petäjävesi), it can have spatially broader effect on microbial community, depending on the contribution of wastewater effluent compared to lake discharge.

On Keuruu study site, the amount of unique OTUs, i.e., OTUs that were not found from the other sampling points, was highest at the wastewater discharge point (545 OTUs, 11.2% of all, Supplementary Figure S2), whereas in Petäjävesi, the abundance of unique OTUs at the wastewater discharge point was similar to the other sampling points (370, 19% of all, Supplementary Figure S2). Using SourceTracker method, the origins of the microbes from wastewater-influenced sampling points were predicted to belong to the natural lake community, to the WWTP community, and to an unknown source that represents the certain microbial taxa which are favored by the wastewater effluent. In Keuruu, 6% of the microbes originated from the WWTP, 43% from the lake natural communities, and 51% were tracked as unknown source (i.e., wastewater favored microbes) at the wastewater discharge point (Supplementary Figure S3). When moving downstream, the proportions of both WWTP-originating and favored microbes were seen to decrease (WWTP-origin: 1% at the 100 m point and < 1% at 200 m point; wastewater favored: 33% at the 100 m point and 30% at 200 m point), whereas the proportion of lake natural microbes increased rapidly (66 and 69%). In Petäjävesi, the proportion of microbes originating from WWTP (2%) was not as significant as in Keuruu. However, the proportion of microbes originating from unknown source was high (90%) at the wastewater discharge point (Supplementary Figure S3). At the downstream points, the microbes originating from WWTP were almost missing (proportion < 1%), while the proportion of wastewater-favored microbes decreased (80% at the 100 m point and 54% at 250 m point) and natural lake microbes increased slowly (19 and 45%). Although the high amount of unique OTUs at the wastewater discharge point was observed only in Keuruu, Source Tracker results demonstrate that on both study sites, a significant proportion of OTUs observed at the wastewater discharge sampling point are not naturally found from the lake sediment communities, but are brought from the WWTP. Our results strongly support the previous findings on WWTPs modifying microbial communities not only through altering environmental conditions, but also through importing microbes to the receiving ecosystems (Chahinian et al., 2012; Drury et al., 2013). However, again, these results reflect the differences in the mixing patterns between these two study sites. If wastewater is freely mixed with lake water (as in Petäjävesi), microbes coming from the WWTP probably cannot survive and establish, as has been previously described in river systems (Garnier et al., 1992). In those environments, the effect of wastewater on sediment microbes seems to be driven through increased nutrient and carbon availability (Chahinian et al., 2012), seen as notably high proportion of wastewater-favored microbes (unknown source) at the Petäjävesi wastewater-influenced sampling points. If wastewater is discharged straight to the sediment surface (as in Keuruu), bacteria have higher probability to settle in and affect the microbial community structure and function.

**FIGURE 4:**
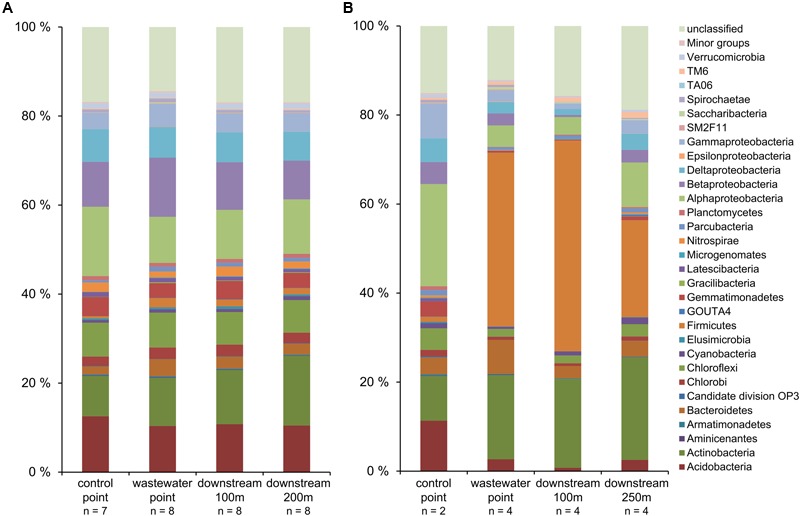
**The relative abundances of bacteria taxa (>1% of relative abundance in at least one sample) (A)** on Keuruu and **(B)** on Petäjävesi study site.

The relative abundances of phylogenetic groups across different sampling points in Keuruu and Petäjävesi were visualized with barplots (**Figure 4**). On both study sites, certain bacterial groups (*Aminicenantes, Bacteroidetes, Epsilonproteobacteria, Firmicutes, Gracilibacteria, Saccharibacteria*) were most abundant at the wastewater discharge sampling point. However, the two study sites had also clear differences in the phylogenetic group abundances. On Keuruu study site, the relative abundance of *Delta-* and *Alphaproteobacteria* decreased, whereas *Beta*- and *Gammaproteobacteria* increased at the wastewater discharge point as compared to control point (**Figure 4**). Overall, *Proteobacteria* were the most common found bacterial phyla, covering on average 32% of the sample sequences in Keuruu data. This was not seen in Petäjävesi, where *Firmicutes* was clearly the most abundant group and the relative abundance of *Proteobacteria* decreased at the wastewater-influenced sampling points. The physiologies of the 20 most abundant OTUs were also clearly distinct between Keuruu and Petäjävesi. In Keuruu, the most common OTUs were mainly aerobic and their relative abundances were rather equal (Supplementary Table S3). In Petäjävesi, anaerobic *Turicibacter* covered 7–21% of OTUs at the wastewater-influenced sampling points and in general, the common OTUs were anaerobic (Supplementary Table S4). The communities (data not shown) as well as the relative abundance of the 20 most common OTUs clearly differed between Keuruu and Petäjävesi WWTPs (Supplementary Table S5).

From the most abundant phylogenetic groups found in this study, *Bacteroidetes* and *Firmicutes*, and *Proteobacteria* in general, have been previously found from WWTPs and wastewater-influenced environments (Zhang et al., 2012; Drury et al., 2013; Lu and Lu, 2014). The high abundance of *Proteobacteria* observed in Keuruu has also been found to be typical for wastewater environments (Zhang et al., 2012; Lu and Lu, 2014). In Petäjävesi, however, the proportion of *Proteobacteria* decreased under wastewater influence, as has been previously observed in river sediments (Drury et al., 2013). This can be explained by differences in the organic matter content and oxygen levels between the two study sites. In Petäjävesi, organic matter concentration was higher and oxygen concentration lower as compared to Keuruu, suggesting that the conditions in Petäjävesi might favor fermentative bacterial groups such as *Clostridium* (Atashgahi et al., 2015), which covered 28% of the *Firmicutes* there. The difference in redox conditions between sites was seen also in the physiology of the 20 most abundant OTUs. In Keuruu, the common OTUs at the wastewater-influenced sampling points were aerobic, whereas in Petäjävesi, they were anaerobic and fermentative. Overall, it is not surprising that we found such large differences in the relative abundances of phylogenetic groups between Keuruu and Petäjävesi sites, as already the WWTPs had distinct microbial communities. Previously, microbial communities have shown to vary significantly from WWTP to another, depending on the wastewater characteristics and origin, and on WWTP treatment configuration (Lu et al., 2014). Furthermore, the proportion of wastewater effluent as compared to the volume and retention time of the receiving waterbody varied between our study sites. This was seen as higher water NO_x_^-^ concentrations as compared wastewater discharge NO_x_^-^ concentrations in Petäjävesi than in Keuruu (Supplementary Table S1), and can partly explain the observed differences in the phylogenetic abundance patterns between the two sites.

### The Effects of Wastewater on N Transformation Potential

**FIGURE 5:**
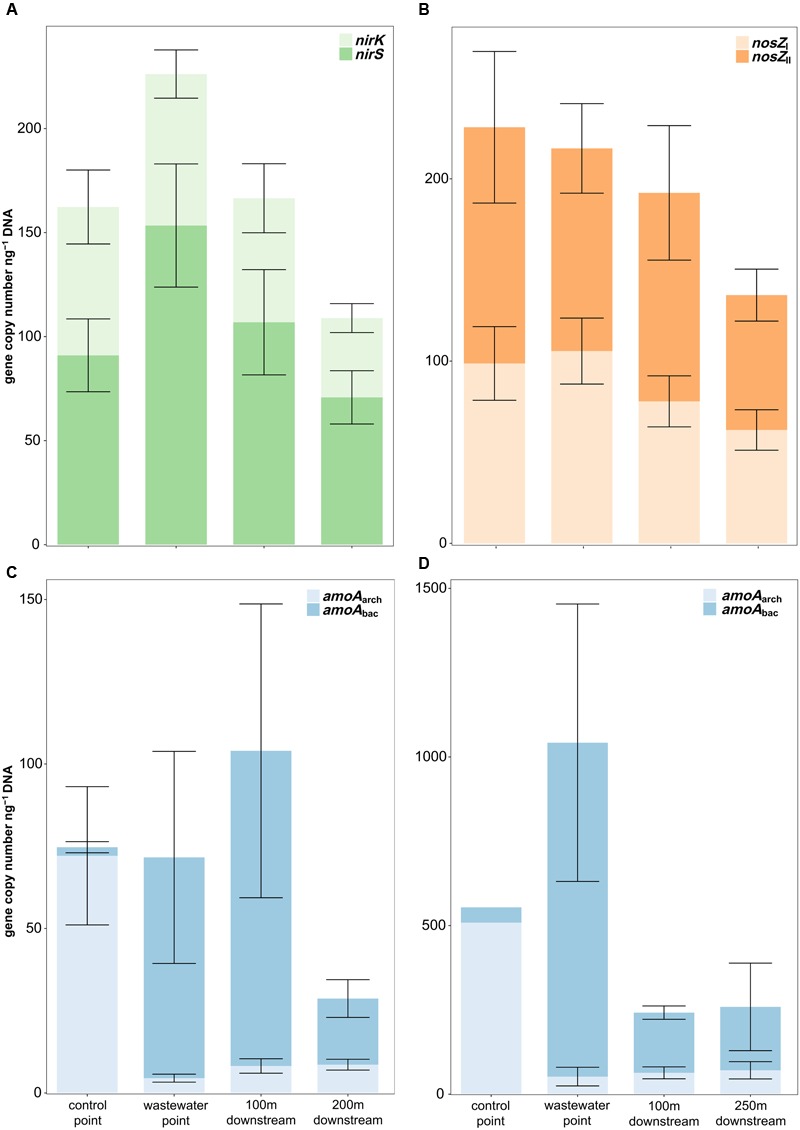
**Relative copy numbers of (A)** *nirS* and *nirK*, **(B)** *nosZ*_I_ and *nosZ*_II_, **(C)** *amoA*_arch_ and *amoA*_bac_ in Keuruu, and **(D)** *amoA*_arch_ and *amoA*_bac_ in Petäjävesi.

On both study sites, the genetic nitrification potential was clearly affected by the wastewater effluent (**Figure 5**). In Keuruu, the nitrifying population was dominated by the AOA (*amoA*_arch_) at the control point (AOA:AOB 28:1), whereas the opposite was seen at the wastewater-influenced sampling points, where the AOB (*amoA*_bac_) became more abundant (AOA:AOB 1:2–1:15; Kruskal–Wallis test, AOA: *H* = 17.4, *P* < 0.001, AOB: *H* = 16.5, *P* < 0.001; **Figure 5**). Similarly, the AOA dominated the nitrifying population at the control point in Petäjävesi (AOA:AOB 11:1), but there the copy numbers of the AOBs were extremely high only at the wastewater discharge point (AOA:AOB 1:19) and decreased rapidly at the downstream points (AOA:AOB 1:3; **Figure 5**). The AOA abundance remained similar throughout the year, but there was some seasonal variation in the AOB gene copy numbers. In Keuruu, the AOB abundance was highest in winter and lowest in summer, whereas in Petäjävesi, the highest AOB copy numbers were recorded in spring. However, a similar trend in the AOB abundance between the wastewater-influenced and the natural sampling points prevailed irrespective of the season. The genetic denitrification potential (copy numbers of *nirS, nirK, nosZ*_I_, and *nosZ*_II_) was measured on Keuruu study site, and did not differ between the sampling points (Kruskal–Wallis test, *P* > 0.05; **Figure 5**). Furthermore, no clear niche separation between *nirS* and *nirK* –harboring organisms or between *nosZ*_I_ and *nosZ*_II_ –harboring organisms was seen between the control point and the wastewater-influenced sampling points (Kruskal–Wallis tests, *P* > 0.05). All denitrification genes were most abundant in winter and in autumn.

In Keuruu, only *amoA*_arch_ abundance correlated with denitrification process rates and N species (**Figure 3**). The abundance of *amoA*_arch_ was lower when total denitrification rate (D14), the proportion of denitrification of the NO_3_^-^ in the water above the sediment (Dw%), NO_x_^-^ or NH_4_^+^ were higher, or when the proportion of coupled nitrification-denitrification (Dn%) was lower (**Figure 3**). The abundance of *nirS* was lower in higher temperatures, whereas *nirS, nosZ*_I_ and *amoA*_bac_ increased with oxygen. All gene abundances increased with the proportion of sediment organic matter (LOI%; **Figure 3**).

Our finding on wastewater-driven changes in the genetic nitrification potential is intriguing, as the end N product after the final nitrification process in both of the WWTPs is supposed to be nitrate (approximately 95% the total N load), which should favor denitrification instead of nitrification. However, ammonium concentrations in the discharged wastewater are still rather high compared to the natural ones, and can even exceed nitrate concentrations, if nitrification process is not fully operating, like seen in Keuruu WWTP in winter 2015. In general, seasonal factors seem to have a minor role in controlling the genetic nitrification potential, as previous studies have already demonstrated that N species and lake trophic status are the key determinants of AOA and AOB dynamics (e.g., Vissers et al., 2013; Bollmann et al., 2014). We suggest that the clear niche difference of AOA and AOB observed between the control sampling point and wastewater-influenced sampling points in this study can be explained by two mechanisms. First, the stable and N poor environment at the control point could be more beneficial for the AOA, while turbulent N rich environment at the wastewater sampling point could support the AOB (Laanbroek et al., 2013; Bollmann et al., 2014). This is supported with the negative correlation found between the AOA abundance and inorganic N concentrations and denitrification processes, which are higher at the wastewater sampling points. Second, a large proportion of AOB might originate from the WWTP, where they are usually the main drivers of the nitrification process (Gao et al., 2014). In the nitrification pools of our study WWTPs, the ratio between AOA and AOB was 1:256 in Keuruu WWTP and 1:9 in Petäjävesi WWTP (data not shown), which indicates that high AOB abundance at the discharge point might originate from the wastewater effluent. We think that the occurrence of AOA is best explained by the first mechanism, as the AOA was almost missing from all wastewater-influenced sampling points both in Keuruu and in Petäjävesi. However, the occurrence of AOB is likely driven by both mechanisms. Especially in Keuruu, the high overall proportion of WWTP-originating bacteria (based on SourceTracker), as well as high proportion of common nitrifiers (*Betaproteobacteria* and *Gammaproteobacteria*; Ferrera and Sánchez, 2016) at the wastewater-influenced points, support the latter mechanism. In Petäjävesi, the high AOB abundance might be more related to wastewater-driven changes in habitat characteristics, as the proportion of WWTP-originating bacteria and common nitrifiers was smaller and the proportion of wastewater-favored microbes was higher than in Keuruu.

Our results indicate that wastewater does not significantly alter the genetic denitrification potential or drive niche separation. We did not find denitrification gene abundances correlating with denitrification process rates or N species, which both follow wastewater gradient, but rather with some general environmental factors (temperature, oxygen). This suggests that the genetic denitrification potential is mainly controlled by seasonal conditions. It is possible that there was some seasonally varying environmental factor (e.g., easily degradable algal carbon, phosphorus, copper, iron; Abell et al., 2010; Black et al., 2016; Myrstener et al., 2016; Robertson and Thamdrup, 2017), which was more limiting that nitrate, explaining the observed seasonality in the genetic denitrification potential. However, the abundances of all denitrification genes correlated with organic matter (LOI%), which was generally higher at the wastewater-influenced sampling points, suggesting that wastewater could also partly promote denitrification gene abundances. Overall, it is not surprising that the genetic denitrification potential is not related to denitrification process rates, since denitrification is a complex process catalyzed by diverse organisms and functional gene abundances are known to explain denitrification process rates rather poorly (Graham et al., 2016). Some microbes can possess only truncated version of denitrification pathway, where the end-product is N_2_O (Graf et al., 2014), which was excluded from denitrification measurements in this study. Furthermore, some denitrification enzymes (*nirK* and *nosZ*_II_) have even been found from non-denitrifying organisms having some other N transformation pathway with other end-product than N_2_ (e.g., DNRA; Sanford et al., 2012), which might explain our results.

Altogether, the effect of wastewater on lake N budget can be beneficial, as it promotes AOB which are considered as major contributors to ammonia oxidation especially in ammonium-rich environments (e.g., Jia and Conrad, 2009; Zhang et al., 2015) and the nitrate produced can then fuel sediment denitrification and enhance the removal of reactive N from the system. However, by promoting AOB abundance, wastewater can also promote “ecosystem disservice,” as the AOB communities were recently found to produce more powerful greenhouse gas N_2_O as a by-product in nitrification than the AOA communities (Hink et al., 2016).

## Conclusion

Here, we showed that the effects of purified and nitrified wastewater on lake sediment microbiology are diverse and dependent on the wastewater discharge settings. Our results suggest that wastewater effluent shapes sediment microbiology not only by providing more nitrate and degrading organic material, but also by bringing new microbes to the lake. We found evidence that the spatial influence of wastewater is more pronounced when it is discharged through a traditional pipeline and is freely mixed with the lake water than when it is directed to the sediment surface throughout the year. This suggests that wastewater effluents can have significant effects on sediment microbiology especially in the nutrient-poor boreal environments, where lakes commonly undergo seasonal mixing. Furthermore, our results imply that wastewater-driven changes in microbial function could possibly impact carbon and nutrient dynamics, and could even promote greenhouse gas emissions. However, to gain a more thorough mechanistic understanding on how wastewater modifies sediment microbial communities and especially those involved in N-cycling, future field-based studies should be complemented with experimental sediment incubations, functional gene sequencing and quantitative reverse transcription PCR (RT-qPCR).

In addition to nutrients and beneficial microbes, wastewater can also bring pathogens and harmful substances, e.g., pollutant or drug residues to the receiving waterbodies. Those could be recognized and quantified by qPCR using probes targeted on pathogen DNA and on the genes involved in the degradation of the harmful substances, similarly as we here targeted on the nitrifying and denitrifying genes. In general, the targeted qPCR approach might be more sensitive than the traditional methods (e.g., cell culturing, high-performance liquid chromatography) and could thus supplement microbial water quality monitoring when estimating the overall impacts of wastewater effluents on surface waters.

## Author Contributions

JS, SA, AR, and MT conceived the project. JS and SA collected the samples, analyzed and interpreted the data. JS, SA, AR, and MT wrote the paper.

## Conflict of Interest Statement

The authors declare that the research was conducted in the absence of any commercial or financial relationships that could be construed as a potential conflict of interest.
