# Supplementary material for: Microbial Community Response on Wastewater Discharge in Boreal Lake Sediments
Source: Front Microbiol. 2017 Apr 25;8:750. doi: 10.3389/fmicb.2017.00750 (PMC5403825; doi:10.3389/fmicb.2017.00750)
Supplement: Supplementary file 1 [file Data_Sheet_1.DOCX]

***Supplementary material***

**Microbial community response on waste water discharge in boreal lake sediments**

**Jatta Saarenheimo^1^**^‡^**, Sanni L. Aalto^1*^**^‡^**, Antti J. Rissanen^2^ and Marja Tiirola^1^**

^1^ University of Jyväskylä, Department of Biological and Environmental Science, P.O.Box 35, 40014 Jyväskylä, Finland

^2^ Laboratory of Chemistry and Bioengineering, Tampere University of Technology, P.O. Box 527, FI-33101 Tampere, Finland

*** Correspondence:** Sanni Aalto, University of Jyväskylä, Department of Biological and Environmental Science, P.O.Box 35, 40014 Jyväskylä, Finland, email: [sanni.lh.aalto@jyu.fi](mailto:sanni.lh.aalto@jyu.fi)

‡These authors contributed equally to this work.

**SUPPLEMENTARY MATERIAL**

**Supplemental Figure S1 |** Non-metric multidimensional scaling (NMDS) of microbial community composition based on Bray-Curtis dissimilarities on Keuruu study site. Four sampling sites are labelled according to legend and vectors indicate fitted environmental factors or N process rates, which correlate significantly (P <0.05) with NMDS coordinates. NO_x_: nitrate concentration; D14: total denitrification rate

**Supplemental Figure S2 |** Venn diagrams showing unique and shared OTUs at four sampling points A) on Keuruu study site and B) on Petäjävesi study site

**Supplemental Figure S3 |** The contributions (average ± SE) of wastewater-favored microbes (unknown), microbes coming from wastewater treatment plant (WWTP), and natural lake microbes to the sediment microbial community predicted using SourceTracker at the wastewater discharge point and at the downstream sampling points A) on Keuruu study site and B) on Petäjävesi study site.

**Supplemental Table 1.** Ammonium and nitrate concentrations discharged from the wastewater treatment plant (WWTP), ammonium, nitrate and oxygen concentrations measured above the lake sediment, the proportion of organic matter of the lake sediment (LOI%) and total denitrification rate (D14) at the wastewater discharge sampling point and at control sampling point on Keuruu ja Petäjävesi study sites in 2015.

**Supplemental Table S2 |** Bacterial diversity (Inverse Simpson index) and richness (chao richness estimator) (mean ± SE) at the control point, wastewater discharge point and downstream sampling points on Keuruu study site. Kruskal Wallis test results are represented.

**Supplemental Table S3 |** 20 most abundant OTUs classification, relative abundances and physiology on Keuruu study site

**Supplemental Table S4 |** 20 most abundant OTUs classification, relative abundances and physiology on Petäjävesi study site

**Supplemental Table S5 |** 20 most abundant OTUs classification, relative abundances and physiology in the Keuruu and Petäjävesi WWTPs


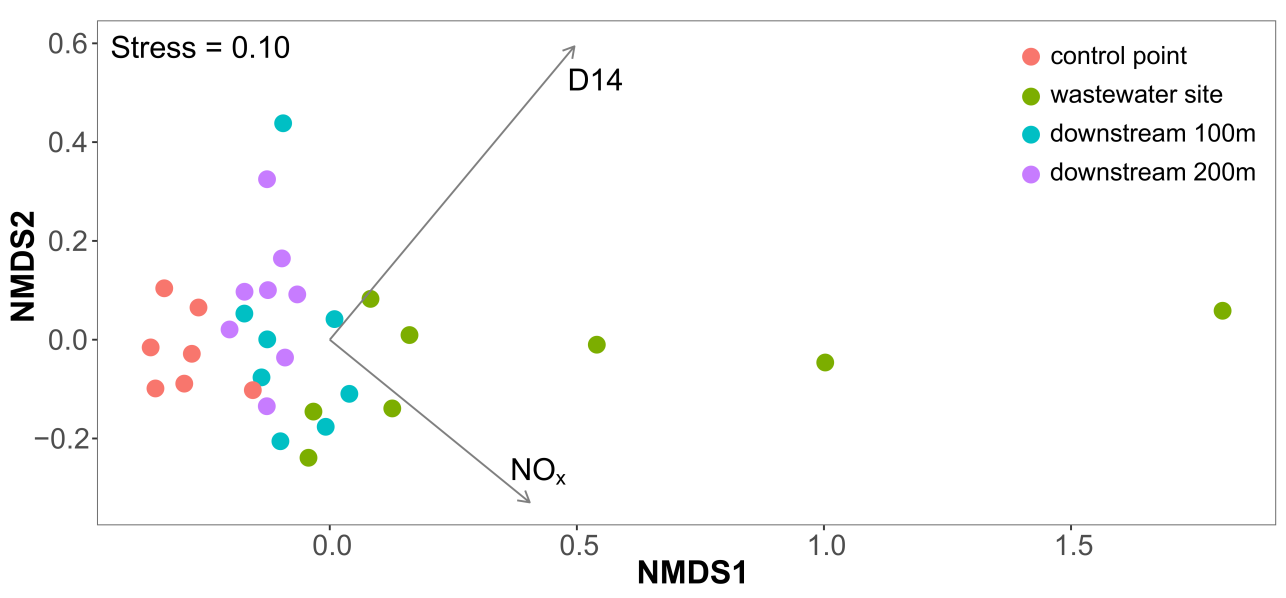


**Supplemental Figure S1 |** Non-metric multidimensional scaling (NMDS) of microbial community composition based on Bray-Curtis dissimilarities on Keuruu study site. Four sampling sites are labelled according to legend and vectors indicate fitted environmental factors or N process rates, which correlate significantly (P <0.05) with NMDS coordinates. NO_x_: nitrate concentration; D14: total denitrification rate


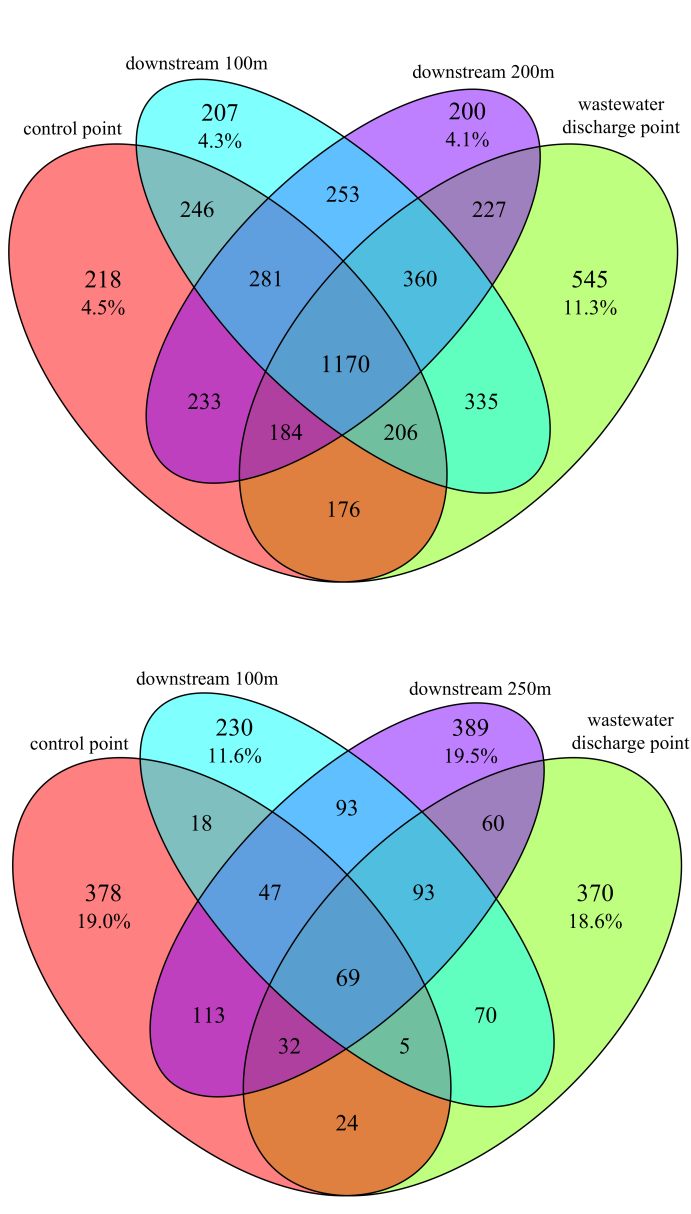


A

B

**Supplemental Figure S2 |** Venn diagrams showing unique and shared OTUs at four sampling points A) on Keuruu study site and B) on Petäjävesi study site


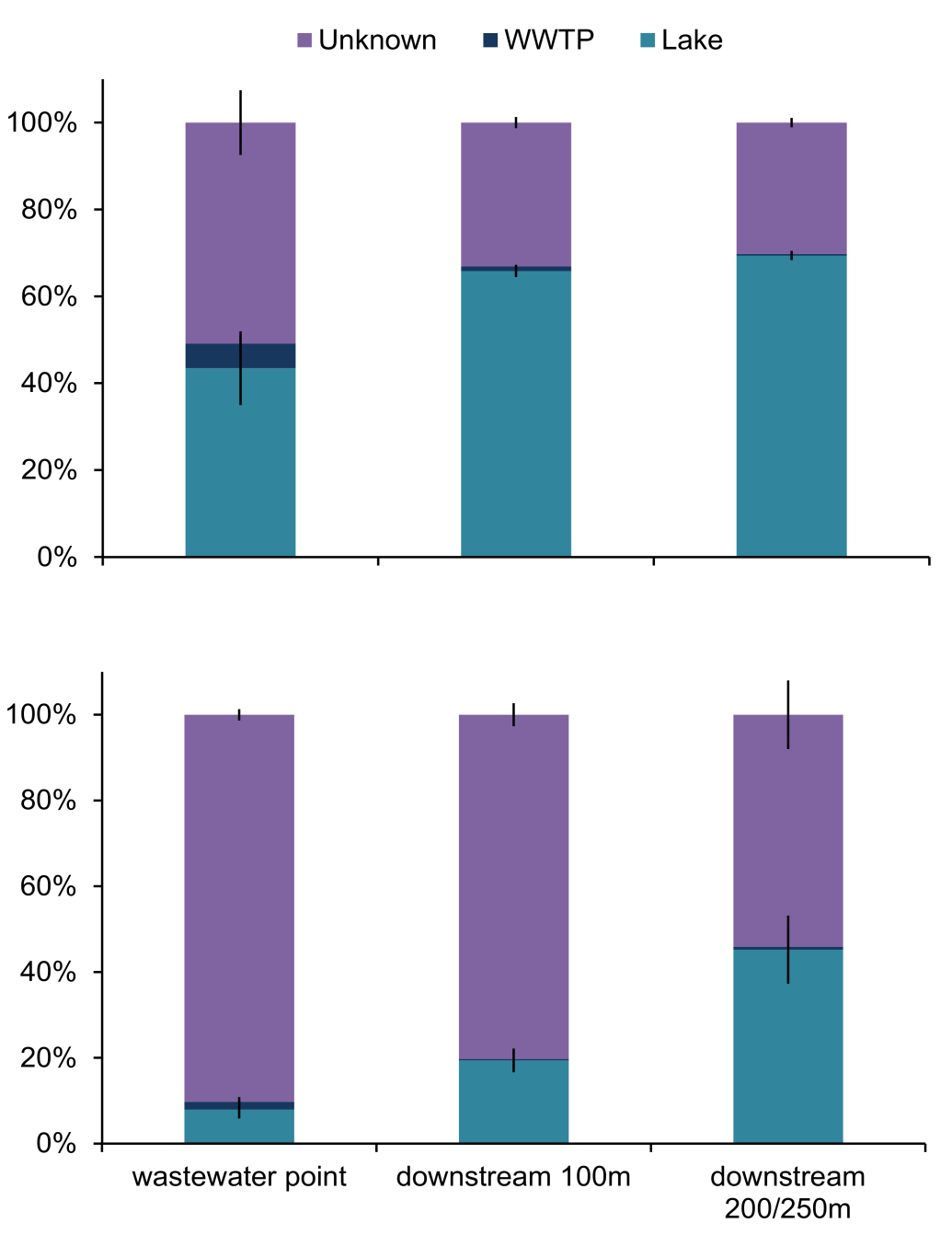


A

B

**Supplemental Figure S3 |** The contributions (average ± SE) of wastewater-favored microbes (unknown), microbes coming from wastewater treatment plant (WWTP), and natural lake microbes to the sediment microbial community predicted using SourceTracker at the wastewater discharge point and at the downstream sampling points A) on Keuruu study site and B) on Petäjävesi study site.

**Supplemental Table 1.** Ammonium and nitrate concentrations discharged from the wastewater treatment plant (WWTP), ammonium, nitrate and oxygen concentrations measured above the lake sediment, the proportion of organic matter of the lake sediment (LOI%) and total denitrification rate (D14) at the wastewater discharge sampling point and at control sampling point on Keuruu ja Petäjävesi study sites in 2015.

|  |  | WWTP | | wastewater discharge point | | | | | control point | | | | |
| --- | --- | --- | --- | --- | --- | --- | --- | --- | --- | --- | --- | --- | --- |
|  |  | NH_4_ | NO_3_ | NH_4_ | NO_x_ | O_2_ | LOI | D14 | NH_4_ | NO_x_ | O_2_ | LOI | D14 |
|  |  | (µmol/L) | (µmol/L) | (µmol/L) | (µmol/L) | mg/L | % | µmol N m^-2^ d^-1^ | (µmol/L) | (µmol/L) | mg/L | % | µmol N m^-2^ d^-1^ |
| Keuruu | winter | 1928 | 671 | 130 | 69 | 12.7 | 12.2 | 2258 | 9 | 12 | 13.9 | 10.4 | 155 |
|  | spring | BD | 1571 | 4 | 18 | 10.8 | 8.4 | 1039 | 5 | 15 | 11.3 | 8.8 | 131 |
|  | summer | 75.0 | 2392 | 4 | 60 | 7.1 | 6.8 | 1595 | 3 | 5 | 7.3 | 8.8 | 265 |
|  | autumn | 132 | 3356 | 9 | 34 | 10.4 | 12.8 | 1463 | 3 | 10 | 10.6 | 9.2 | 220 |
| Petäjävesi | winter | BD | 2356 | 8 | 160 | 9.3 | 20.6 | 3923 | 10 | 12 | 11.4 | 8.4 | 107 |
|  | spring | 12.1 | 1784 | 6 | 89 | 8.7 | n/a | 3268 | 4 | 9 | 9.5 | n/a | 126 |

BD = below detection limit

**Supplemental Table 2.** Bacterial diversity (Inverse Simpson index) and richness (chao richness estimator) (mean ± SE) at the control point, wastewater discharge point and downstream sampling points on Keuruu study site. Kruskal Wallis test results are represented.

|  | Inverse Simpson | | | chao | | |
| --- | --- | --- | --- | --- | --- | --- |
| control point | 244 | ± | 15 | 1930 | ± | 51 |
| wastewater point | 245 | ± | 28 | 1892 | ± | 202 |
| downstream 100m | 251 | ± | 39 | 2097 | ± | 87 |
| downstream 200m | 187 | ± | 24 | 2032 | ± | 50 |
| Kruskal Wallis | H= 3.62, P = 0.31 | | | H= 3.48, P = 0.32 | | |

**Supplemental Table S3 |** 20 most abundant OTUs classification, relative abundances and physiology on Keuruu study site

|  | **Relative abundance (%)^a^** | | | |  |  |  |
| --- | --- | --- | --- | --- | --- | --- | --- |
| **OTU** | **ctrl point** | **ww site** | **100m** | **250m** | **Classification^b^** | **Physiology** | **References** |
| Otu00001 | 2.32 | 2.14 | 3.90 | 5.39 | *Gaiellaceae* | aerobic chemo-organotroph | Albuquerque and da Costa 2014 |
| Otu00005 | 1.04 | 1.94 | 1.75 | 1.23 | *Betaproteobacteria* | chemo-organotroph/chemolithotroph/N2-fixing | Garrity et al., 2005 |
| Otu00009 | 1.84 | 0.72 | 1.17 | 0.96 | *Variibacter* | aerobic heterotroph | Kim et al., 2014 |
| Otu00006 | 1.40 | 0.63 | 1.12 | 1.26 | *Methylocystaceae* | type II aerobic methanotroph | Webb et al., 2014 |
| Otu00017 | 0.76 | 0.75 | 1.12 | 1.05 | *Deltaproteobacteria* | aerobic or anaerobic heterotroph | Garrity et al., 2005 |
| Otu00012 | 0.72 | 0.81 | 1.07 | 1.05 | *Methylococcales* | type I aerobic methanotroph | Webb et al., 2014 |
| Otu00015 | 1.00 | 0.47 | 0.74 | 1.37 | *Actinobacteria* | - |  |
| Otu00007 | 0.78 | 0.56 | 0.91 | 1.19 | *unclassified* | - |  |
| Otu00010 | 0.76 | 0.40 | 1.05 | 1.21 | *unclassified* | - |  |
| Otu00016 | 0.74 | 1.02 | 0.67 | 0.96 | *Bradyrhizobiaceae* | N2-fixation/photosynthesis/aerobic and anaerobic respiration | de Souza et al., 2014 |
| Otu00013 | 0.28 | 0.51 | 0.91 | 1.24 | *Gaiellales* | aerobic chemo-organotroph | Albuquerque and da Costa 2014 |
| Otu00019 | 0.84 | 0.74 | 0.72 | 0.63 | *Pseudolabrys* | aerobic heterotroph | Kämpfer et al., 2006 |
| Otu00018 | 0.66 | 0.46 | 0.68 | 1.05 | *Rhizobiales* | N2-fixation/photosynthesis/aerobic and anaerobic respiration | de Souza et al., 2014 |
| Otu00021 | 1.06 | 0.51 | 0.60 | 0.61 | *Betaproteobacteria* | chemo-organotroph/chemolithotroph/N2-fixing | Garrity et al., 2005 |
| Otu00011 | 0.58 | 0.28 | 0.74 | 1.07 | *Mycobacterium* | heterotrophs | Lory 2014 |
| Otu00022 | 0.56 | 0.56 | 0.81 | 0.58 | *Betaproteobacteria* | chemo-organotroph/chemolithotroph/N2-fixing | Garrity et al., 2005 |
| Otu00024 | 0.62 | 0.40 | 0.75 | 0.67 | *Betaproteobacteria* | chemo-organotroph/chemolithotroph/N2-fixing | Garrity et al., 2005 |
| Otu00023 | 1.54 | 0.14 | 0.35 | 0.44 | *Xanthobacteraceae* | aerobic heterotroph | Kim et al., 2014 |
| Otu00026 | 0.56 | 0.53 | 0.60 | 0.56 | *Chloroflexi* | anoxygenic phototroph | Hanada, 2014 |
| Otu00028 | 1.38 | 0.18 | 0.40 | 0.30 | *Betaproteobacteria* | chemo-organotroph/chemolithotroph/N2-fixing | Garrity et al., 2005 |

^a^each data point is a mean value (of seven samples at the control point and of eight samples at the wastewater sampling points)

^b^classification is based on SILVA least common ancestor

**Supplemental Table S4 |** 20 most abundant OTUs classification, relative abundances and physiology on Petäjävesi study site

|  | **Relative abundance (%)^a^** | | | |  |  |  |
| --- | --- | --- | --- | --- | --- | --- | --- |
| **OTU** | **ctrl point** | **ww site** | **100m** | **250m** | **Classification^b^** | **Physiology** | **References** |
| Otu00002 | 0.35 | 17.68 | 21.04 | 7.11 | *Turicibacter* | anaerobic chemo-organotroph | Bosshard et al., 2002 |
| Otu00004 | 0.21 | 6.16 | 7.18 | 3.36 | *Intestinibacter* | anaerobic heterotroph | Gerritsen et al., 2014 |
| Otu00008 | 0.14 | 4.76 | 6.30 | 2.80 | *Clostridium* | fermentative heterotroph | Kallistova et al., 2014 |
| Otu00014 | 0.00 | 4.31 | 5.08 | 2.59 | *Clostridium* | fermentative heterotroph | Kallistova et al., 2014 |
| Otu00001 | 1.19 | 0.77 | 3.05 | 3.71 | *Gaiella* | aerobic chemo-organotroph | Albuquerque and da Costa 2014 |
| Otu00007 | 0.98 | 0.74 | 2.17 | 3.57 | *unclassified* | - |  |
| Otu00011 | 1.19 | 0.81 | 2.21 | 3.29 | *Mycobacterium* | heterotroph | Lory 2014 |
| Otu00033 | 0.07 | 1.96 | 2.49 | 0.88 | *Terrisporobacter* | anaerobic heterotroph | Gerritsen et al., 2014 |
| Otu00006 | 3.85 | 0.25 | 0.77 | 2.07 | *Methylocystis* | type II aerobic methanotroph | Webb et al., 2014 |
| Otu00013 | 0.49 | 0.56 | 1.89 | 2.28 | *Gaiellales* | aerobic chemo-organotroph | Albuquerque and da Costa 2014 |
| Otu00046 | 0.00 | 1.89 | 2.03 | 0.84 | *Clostridium* | fermentative heterotroph | Kallistova et al. ,2014 |
| Otu00010 | 1.54 | 0.74 | 1.33 | 1.86 | unclassified | - |  |
| Otu00044 | 0.35 | 1.79 | 1.58 | 1.02 | unclassified | - |  |
| Otu00020 | 2.17 | 0.53 | 0.91 | 1.93 | *Methylocystis* | type II aerobic methanotroph | Webb et al., 2014 |
| Otu00061 | 0.21 | 0.35 | 1.05 | 1.54 | *Actinobacteria* | - |  |
| Otu00092 | 0.70 | 0.25 | 0.95 | 1.19 | *Actinobacteria* | - |  |
| Otu00066 | 0.14 | 1.58 | 0.49 | 0.56 | *Galbitalea* | aerobic heterotroph | Kim et al., 2014 |
| Otu00047 | 0.07 | 1.33 | 0.60 | 0.32 | *Candidatus_Microthrix* | aerobic chemoorganotroph | Rossetti et al., 2005 |
| Otu00083 | 0.00 | 0.60 | 0.77 | 0.81 | *Intestinibacter* | anaerobic heterotroph | Gerritsen et al., 2014 |
| Otu00088 | 0.14 | 1.40 | 0.28 | 0.32 | *Ferruginibacter* | aerobic heterotroph | Lim et al., 2009 |

^a^each data point is a mean value (of two samples at the control point and of four samples at the wastewater sampling points)

^b^classification is based on SILVA least common ancestor

**Supplemental Table S5 |** 20 most abundant OTUs classification, relative abundances and physiology in the Keuruu and Petäjävesi WWTPs

|  | **Relative abundance (%)** | | | |  |  |  |
| --- | --- | --- | --- | --- | --- | --- | --- |
| **OTU** | Keuruu WWTP nitrification pool^a^ | Keuruu WWTP discharge | Petäjävesi WWTP nitrification pool | Petäjävesi WWTP discharge | **Classification^b^** | **Physiology** | **References** |
| Otu00003 | 0.28 | 5.74 | 35.01 | 74.37 | *Betaproteobacteria* | chemo-organotroph/chemolithotroph/N2-fixing | Garrity et al., 2005 |
| Otu00032 | 0.77 | 0.28 | 17.37 | 7.56 | *Gammaproteobacteria* | aerobic and anaerobic autotroph/heterotroph | Garrity et al., 2005 |
| Otu00051 | 1.47 | 23.81 | 0.00 | 0.00 | *Betaproteobacteria* | chemo-organotroph/chemolithotroph/N2-fixing | Garrity et al., 2005 |
| Otu00027 | 5.95 | 3.36 | 0.00 | 0.00 | *Ferribacterium* | aerobic chemo-organotroph | Oren, 2014 |
| Otu00025 | 4.62 | 1.54 | 0.56 | 0.14 | *Comamonadaceae* | chemo-organotroph/N2-fixing/photoautotroph/fermentative | Willems 2014 |
| Otu00101 | 4.62 | 1.68 | 0.28 | 0.00 | *Byssovorax* | aerobic heterotroph | Garcia and Müller, 2014a |
| Otu00104 | 2.31 | 0.28 | 4.90 | 0.42 | *Flavobacterium* | aerobic chemo-organotroph | McBride et al., 2014a |
| Otu00105 | 3.01 | 2.10 | 0.56 | 0.42 | *Alkanindiges* | aerobic heterotroph | Bogan et al., 2003 |
| Otu00129 | 1.05 | 5.74 | 0.28 | 0.00 | *Albidiferax* | anaerobic chemo-organotroph | Willems 2014 |
| Otu00123 | 2.80 | 1.82 | 0.00 | 0.00 | *Arenimonas* | aerobic heterotroph | Kwon et al., 2007 |
| Otu00153 | 2.24 | 1.12 | 0.00 | 0.00 | *unclassified* | - |  |
| Otu00110 | 1.82 | 0.84 | 0.56 | 0.28 | *Methylorosula* | aerobic heterotroph | Berestovskaya et al., 2012 |
| Otu00134 | 1.75 | 0.56 | 0.84 | 0.14 | *Cytophagaceae* | aerobic heterotroph | McBride et al., 2014b |
| Otu00216 | 2.03 | 0.42 | 0.42 | 0.00 | *Nannocystis* | aerobic chemo-organotroph | Garcia and Müller, 2014b |
| Otu00078 | 1.75 | 0.98 | 0.00 | 0.00 | *Actinobacteria* | - |  |
| Otu00333 | 0.98 | 2.24 | 0.00 | 0.00 | *Parcubacteria* | - |  |
| Otu00305 | 1.61 | 0.28 | 0.28 | 0.00 | *Flavobacterium* | aerobic chemo-organotroph | McBride et al., 2014a |
| Otu00088 | 1.05 | 0.70 | 0.84 | 0.00 | *Ferruginibacter* | aerobic heterotroph | Lim et al., 2009 |
| Otu00487 | 0.00 | 0.00 | 1.54 | 2.10 | *unclassified* | - |  |
| Otu00250 | 1.40 | 0.42 | 0.00 | 0.00 | *Bacteroidetes* | - |  |

^a^data point is a mean value of two samples

^b^classification is based on SILVA least common ancestor

**References**

Albuquerque, L., and da Costa, M.S. (2014). “The Family Gaiellaceae” in *The Prokaryotes – Actinobacteria*, eds. E. Rosenberg, E.F. DeLong, S. Lory, E. Stackebrandt, and F. Thompson (Berlin, Springer-Verlag), 357-360.

Berestovskaya, J.J., Kotsyurbenko, O.R., Tourova, T.P., Kolganova, T.V., Doronina, N.V., Golyshin, P.N., and Vasilyeva, L.V. (2012). *Methylorosula polaris* gen. nov., sp. nov., an aerobic, facultatively methylotrophic psychrotolerant bacterium from tundra wetland soil. *Int. J. Syst. Evol. Microbiol.* 62(3), 638-646.

Bogan, B.W., Sullivan, W.R., Kayser, K.J., Derr, K.D., Aldrich, H.C., and Paterek, J.R. (2003). *Alkanindiges illinoisensis* gen. nov., sp. nov., an obligately hydrocarbonoclastic, aerobic squalane-degrading bacterium isolated from oilfield soils. *Int. J. Syst. Evol. Microbiol.* 53, 1389-1395.

Bosshard, P.P., Zbinden, R., and Altwegg, M. (2002). *Turicibacter sanguinis* gen. nov., sp. nov., a novel anaerobic, Gram-positive bacterium. *Int. J. Syst. Evol. Microbiol.* 52(4), 1263-1266.

Garcia, R., and Müller, R. (2014a). “The Family Polyangiaceae” in The Prokaryotes – Deltaproteobacteria and Epsilonproteobacteria, eds. E. Rosenberg, E.F. DeLong, S. Lory, E. Stackebrandt, and F. Thompson (Berlin, Springer-Verlag), 247-279.

Garcia, R., and Müller, R. (2014b). “The Family Nannocystaceae” in *The Prokaryotes – Deltaproteobacteria and Epsilonproteobacteria*, eds. E. Rosenberg, E.F. DeLong, S. Lory, E. Stackebrandt, and F. Thompson (Berlin, Springer-Verlag), 213-229.

Garrity, G., Brenner, D.J., Krieg, N.R., and Staley, J.T. (2005). *Bergey's Manual of Systematic Bacteriology –Volume Two: The Proteobacteria (Part C)*. New York: Springer Publishing.

Gerritsen, J., Fuentes, S., Grievink, W., van Niftrik, L., Tindall, B.J., Timmerman, H.M., Rijkers, G.T., and Smidt, H. (2014). Characterization of *Romboutsia ilealis* gen. nov., sp. nov., isolated from the gastro-intestinal tract of a rat, and proposal for the reclassification of five closely related members of the genus *Clostridium* into the genera *Romboutsia* gen. nov., *Intestinibacter* gen. nov., *Terrisporobacter* gen. nov. and *Asaccharospora* gen. nov. *Int. J. Syst. Evol. Microbiol.* 64(5), 1600-1616.

Hanada, S. (2014). " The Phylum Chloroflexi, the Family Chloroflexaceae, and the Related Phototrophic Families Oscillochloridaceae and Roseiflexaceae" in *The Prokaryotes – Other Major Lineages of Bacteria and the Archaea*, eds. E. Rosenberg, E.F. DeLong, S. Lory, E. Stackebrandt, and F. Thompson (Berlin, Springer-Verlag), 515-532.

Kallistova, A.Y., Goel, G., and Nozhevnikova, A.N. (2014). Microbial diversity of methanogenic communities in the systems for anaerobic treatment of organic waste. *Microbiology* 83(5), 462-483.

Kim, K.K., Lee, K.C., Eom, M.K., Kim, J.S., Kim, D.S., Ko, S.H., Kim, B.H., and Lee, J.S. (2014). *Variibacter gotjawalensis* gen. nov., sp. nov., isolated from soil of a lava forest. *Anton. Leeuw. J. Microb.* 105(5), 915-924.

Kwon, S.W., Kim, B.Y., Weon, H.Y., Baek, Y.K., and Go, S.J. (2007). *Arenimonas donghaensis* gen. nov., sp. nov., isolated from seashore sand. *Int. J. Syst. Evol. Microbiol.* 57(5), 954-958.

Kämpfer, P., Young, C.C., Arun, A.B., Shen, F.T., Jäckel, U., Rossello-Mora, R., Lai, W.A., and Rekha, P.D. (2006). *Pseudolabrys taiwanensis* gen. nov., sp. nov., an alphaproteobacterium isolated from soil. *Int. J. Syst. Evol. Microbiol.* 56(10), 2469-2472.

Lim, J.H., Baek, S.H., and Lee, S.T. (2009). *Ferruginibacter alkalilentus* gen. nov., sp. nov. and *Ferruginibacter lapsinanis* sp. nov., novel members of the family ‘*Chitinophagaceae*’in the phylum Bacteroidetes, isolated from freshwater sediment. *Int. J. Syst. Evol. Microbiol.* 59(10), 2394-2399.

Lory, S. (2014) “The Family Mycobacteriaceae” in *The Prokaryotes – Actinobacteria,* eds. E. Rosenberg, E.F. DeLong, S. Lory, E. Stackebrandt, and F. Thompson (Berlin, Springer-Verlag), 571-575.

McBride, M.J. (2014a). "The family Flavobacteriaceae" in *The Prokaryotes – Other Major Lineages of Bacteria and the Archaea*, eds. E. Rosenberg, E.F. DeLong, S. Lory, E. Stackebrandt, and F. Thompson (Berlin, Springer-Verlag), 643-676.

McBride, M.J., Liu, W., Lu, X., Zhu, Y., and Zhang, W. (2014b). “The family Cytophagaceae” *The Prokaryotes – Other Major Lineages of Bacteria and the Archaea*, eds. E. Rosenberg, E.F. DeLong, S. Lory, E. Stackebrandt, and F. Thompson (Berlin, Springer-Verlag), 577-593.

Oren, A. (2014). “The Family Rhodocyclaceae” in *The Prokaryotes – Alphaproteobacteria and Betaproteobacteria*, eds. E. Rosenberg, E.F. DeLong, S. Lory, E. Stackebrandt, and F. Thompson (Berlin, Springer-Verlag), 975-998.

Rossetti, S., Tomei, M.C., Nielsen, P.H., and Tandoi, V. (2005). “*Microthrix parvicella*”, a filamentous bacterium causing bulking and foaming in activated sludge systems: a review of current knowledge. FEMS Microbiol. Rev. 29(1), 49-64.

de Souza, J.A.M., Alves, L.M.C., de Mello Varani, A., and de Macedo Lemos, E.G. (2014). “The Family Bradyrhizobiaceae” in *The Prokaryotes – Alphaproteobacteria and Betaproteobacteria*, eds. E. Rosenberg, E.F. DeLong, S. Lory, E. Stackebrandt, and F. Thompson (Berlin, Springer-Verlag), 135-154.

Webb, H.K., Ng, H.J., and Ivanova, E.P. (2014). “The Family Methylocystaceae” in *The Prokaryotes – Alphaproteobacteria and Betaproteobacteria*, eds. E. Rosenberg, E.F. DeLong, S. Lory, E. Stackebrandt, and F. Thompson (Berlin, Springer-Verlag), 341-347.

Willems, A. (2014). “The Family Comamonadaceae” in *The Prokaryotes – Alphaproteobacteria and Betaproteobacteria*, eds. E. Rosenberg, E.F. DeLong, S. Lory, E. Stackebrandt, and F. Thompson (Berlin, Springer-Verlag), 777-851.
